# Barriers to and facilitators of timely family consent in caesarean sections: Experiences, perspectives and associated factors–a mixed-methods study in Somaliland

**DOI:** 10.1371/journal.pone.0342475

**Published:** 2026-03-11

**Authors:** Jonah Kiruja, Fatumo Osman, Helena Litorp, Jama Ali Egal, Amina Esse, Marie Klingberg-Allvin

**Affiliations:** 1 School of Health and Welfare, Dalarna University, Falun, Sweden; 2 University of Hargeisa, Hargeisa, Somaliland; 3 Department of Women’s and Children’s Health, Uppsala University, Uppsala, Sweden; 4 Department of Global Public Health, Karolinska Institutet, Stockholm, Sweden; 5 Department of Midwifery, University of Hargeisa, Hargeisa, Somaliland; 6 Department of Women’s and Children’s Health, Karolinska Institutet, Stockholm, Sweden; The University of Melbourne School of Population and Global Health, AUSTRALIA

## Abstract

**Introduction:**

In many countries, the consent for caesarean section (CS), when indicated, is made by the woman herself. However, in Somaliland, the family (husband, father, or other close male family member) are required to make the consent for CS to be performed, a process that can be time-consuming and result in adverse outcomes. This study aimed to investigate the barriers to and facilitators of timely family consent in caesarean sections at the national referral hospital in Somaliland.

**Methodology:**

A convergent mixed-methods study with a parallel sampling method was conducted at the national referral hospital in Somaliland. Quantitative data was collected on timely vs. late family consent for CS, as well as sociodemographic and obstetric characteristics. Data were analysed using binary and multivariable logistic regression. In addition, in-depth interviews were conducted and analysed using thematic analysis.

**Results:**

Of the 516 women included in the quantitative phase of the study, 16 participated in the in-depth interviews. The quantitative results showed that women with hypertensive disorders (aOR 8.491; 95% 1.076–66.991) and obstetric haemorrhage (aOR 3.209; 95% CI 1.159–8.887) had higher odds of late family consent compared to women without hypertensive disorders and obstetric haemorrhage respectively. The themes that emerged on barriers to timely family consent for CS were poor communication and understanding, delayed informed choice for CS, differences in understanding between family members on the indication for CS, and absence of the person providing formal consent. The themes that emerged on facilitators of timely family consent were the husband’s autonomous decision making for CS and adequate disclosure of all relevant information about CS.

**Conclusion:**

A standard counselling package can be designed on educating family members on the importance of timely CS consent during the antenatal period with male involvement. A policy should be developed that gives women the autonomy to make health decisions and give consent in maternal health emergencies. Healthcare providers need training on effective communication when requesting CS consent, with a focus on the elements of informed consent.

## Introduction

To avert maternal morbidity and maternal mortality, women are often required to use emergency obstetric care services such as Caesarean section (CS) [1]. Women’s autonomy in making decisions about maternal health, such as CS, is considered key to improving maternal health outcomes [2]; however, evidence has shown that family involvement in decision-making can also be a vital approach for improving maternal health [3]. In particular, timely family decision-making by giving early consent for emergency CS contributes towards reduced duration between the date and time the family is notified to give consent and the date and time the CS is performed improving maternal and foetal outcomes [4].

To improve the access to CS, the government of Somaliland has been scaling up emergency obstetric care services through establishment of emergency obstetric care health facilities. This included deployment of healthcare providers, equipping the facilities with supplies and equipment [5]. In Somaliland, the country’s public and private sectors are both involved with the delivery of CS services. The public healthcare system is organized into four levels: the primary health unit, maternal and child health centre, regional health centre or referral district hospital, and regional referral hospital [6]. The national referral hospital serves as the main referral hospital serving not only the Maroodi Jeex region where the capital city of Somaliland, Hargeisa, is situated, but also all the other five regions of Somaliland: Sool, Sanaag, Sahil, Awdal, and Togdheer.

The Somaliland national CS rate is estimated at 4%, indicating possible underuse of CS in management of obstetric complications [7]. In Somaliland, the husband, a male family member, or the father to the woman that requires CS gives consent. The woman herself cannot give consent for CS to be performed [8]. Previous studies have shown that healthcare providers face challenges when seeking consent from family members, including the husband to the woman that needs CS [5,8].

In situations where there is a delay from the family in giving consent for CS, women end up with severe maternal outcomes, such as maternal near miss or maternal death, and newborns die or develop severe complications [8,9]. A maternal near miss is when a woman nearly dies but survives during pregnancy, birth, or within 42 days after birth.

Several studies in Somaliland have investigated the reasons for high maternal morbidity and mortality [8,10,11]. These studies have identified that consent is given by the family members such as the woman’s husband and/or male blood relatives rather than the woman herself, which can be time consuming, leading to severe maternal outcomes [5,8]. Moreover, the studies showed that low socioeconomic status and healthcare provider-related factors, such as miscommunication, inadequate interprofessional collaboration, and inadequate infrastructure, contributed to delays in receiving emergency obstetric care. Family decision-making barriers contributed most to delayed provision of emergency obstetric care of greater than 3 hours, which was found to be associated with severe maternal outcomes [12].

Understanding the experiences of timely family consent for CS from the perspective of women who gave birth through CS and identifying associated factors can provide knowledge to develop pragmatic interventions aiming to reduce delays in obtaining consent from family members. In our study, we aimed to investigate the barriers to and facilitators of timely family decision-making about CSs at the national referral hospital in Somaliland.

## Materials and methods

**Fig 1:**
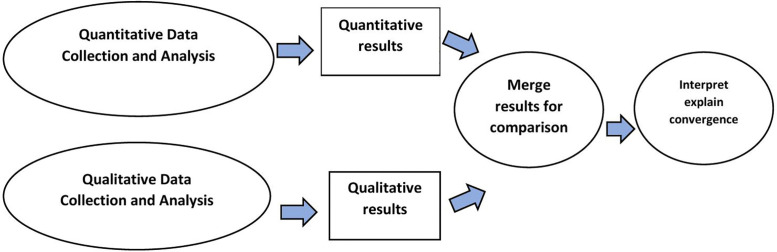
Convergent mixed-methods study design. Quantitative and qualitative data were collected and analyzed separately. Results from both strands were then merged for comparison and interpreted to explain areas of convergence.

In this study, we adopted a convergent mixed-method design [13] combining a quantitative facility-based survey to identify factors associated with timely family decision-making for CS with a qualitative study exploring the barriers to and facilitators of timely family decision-making for CS at the national referral hospital in Somaliland. The convergent mixed-methods design involved collection of different but complementary data on the same topic (Fig 1).

The rationale behind this mixed-methods design was to combine the strengths of both quantitative and qualitative methods in answering the research questions, and provide a deeper understanding of the research problem [13].

The method enabled a broader understanding of the research question in this study by enabling us to investigate barriers to and facilitators of family decision-making about CS from various perspectives of maternal near miss and hence compare and contrast data from quantitative and qualitative methods. A parallel sampling method was used in this study. The study participants recruited in the qualitative phase of the study were drawn from the participants selected in the quantitative phase of the study. Data from both the quantitative and qualitative parts of the study were collected during approximately the same period. After completing analyses of both quantitative and qualitative data separately, data integration was done by following the thread [14] on the barriers to and facilitators of timely family consent about CS.

### Setting

Somaliland is a self-autonomous state with a population of approximately 4.2 million, with 53% residing in urban areas and 47% in rural areas. The adult literacy rate for women aged 15–49 years is 41% [15]. This study was conducted at the national referral hospital in Somaliland situated in Hargeisa City in the Maroodi Jeex region. The national referral hospital provides comprehensive emergency obstetric care, with approximately 6000 women giving birth at the health facility every year, of which approximately 1255 (18.8%) give birth via CS [10].

#### Quantitative study.

*Study design*: The data set in the current study is a subsample of all women who underwent CS (n = 516) within a hospital-based prospective cross-sectional study (n = 6658) conducted to investigate maternal and newborn outcomes among pregnant women in Somaliland described elsewhere [10].

*Study population*: All women who underwent CS at the national referral hospital between the 15^th^ of April 2019 and 30^th^ of March 2020 and whose performance of CS was majorly influenced by the family decision making process were included in the study. Informed by our previous study [5], family consent is a “collective decision-making process in which several decision makers in the extended family structure (father/mother, father/mother in laws, and husband to the woman giving birth) are involved with deliberations on need for CS before the male family member provides formal consent for CS to be performed”. Women whose CS decision was majorly influenced by healthcare providers or financial barriers were excluded. Similarly, women with missing information related to family consent were excluded from the study. The women included were divided into two groups for comparison: women with timely family consent and those with late family consent. *Timely family consent* for CS to be performed was defined as CS performed in less than 3 hours following consent provided by the family, whereas *late family consent* for CS to be performed was defined as CS performed in more than 3 hours following delayed consent from the family members. The cut-off of 3 hours was used based on the findings of our previous study that showed that CS delayed for more than 3 hours was associated with adverse maternal outcomes [12].

International quality standards generally recommend decision-to-incision intervals of 30–75 minutes for emergency cesarean sections [16]. However, such benchmarks are often unachievable in low-resource health systems such as Somaliland, where sociocultural factors notably the need for family consent and structural limitations, including staff shortages and constrained operating theatre capacity, contribute to longer delays [5]. In recognition of these realities, a 3-hour threshold was adopted as a context-specific and evidence-based benchmark, reflecting findings from our prior study in Somaliland [12], while acknowledging that shorter intervals remain optimal for maternal and perinatal safety [17].

*Sample Size and Sampling*: Using Cochran’s formula for sample size calculation, an assumed proportion of 50% (which maximizes variability), a 95% confidence level, and a 5% margin of error, the initial sample size was calculated to be 385. This ensures an adequate representation of the target population while preserving statistical validity. For this study, all the 516 participants were included, which exceeds the minimum required sample size, thereby enhancing the robustness and reliability of the findings.

*Data collection*: A data abstraction form, based on the validated World Health Organization Maternal Near-Miss tool, was used to collect data from medical records upon admission. This standardized tool contains predefined variables assessing sociodemographic factors, obstetric history, and factors associated with family consent [18]. The tool has been widely used in similar studies focusing on emergency obstetric care interventions such as cesarean section, ensuring its validity and reliability [19,20]. Women were followed until discharge, and data were systematically recorded to maintain consistency and accuracy. Trained data collectors comprising a team of midwives, nurses and doctors extracted data from the medical records to the data abstraction form. Periodic quality assurance checks were conducted to ensure that there was no missing data in the forms. For the missing data identified, the data was retrieved from the attached medical record. If data was missing in the medical record, the person responsible with providing care to that woman was contacted to provide the information. The first and fourth author, along with a team of researchers in the maternal health research group entered the collected data into Statistical Package of Social Sciences (SPSS) version 20.

*Variables*: The dependent variables were timely or late performance of CS mainly due to family consent. The independent variables were sociodemographic characteristics such as age, education, residence area, obstetric characteristics such as parity, previous history of CS, underlying causes, hypertensive disorders, obstetric haemorrhage, and contributory/associated factors such as anaemia or obstructed labour/prolonged labour.

*Data analysis*: Univariate, bivariate, and multivariable analyses were performed using SPSS. The Chi-square test was chosen to compare differences between the two groups (timely vs. late family consent for CS) as it is appropriate for categorical data. Bivariate logistic regression was conducted to identify variables significantly associated with timely or late family consent, as this method is suitable for assessing relationships between independent variables and a binary outcome [21]. To control for potential confounders, variables with a p-value of < 0.2 in the bivariate analysis were included in the multivariable model. This threshold is commonly used in regression modeling to ensure that important predictors are not excluded prematurely [22]. The Hosmer–Lemeshow goodness-of-fit test was used to evaluate model adequacy, yielding a p-value of 0.12, indicating that the model provided a good fit to the data (p > 0.05). To control for multiple comparisons, the Bonferroni correction was applied to adjust the significance threshold for the number of tests performed. Since 6 variables were tested, the corrected p-value threshold was set to p ≤ 0.05/ 6 = 0.0083. As such, factors associated with timely or late family consent were considered statistically significant at p ≤ 0.008. This adjustment helps reduce the risk of Type I errors due to multiple testing [23].

#### Qualitative study.

*Study design*: An exploratory study design [24] using in-depth interviews was applied in this study. In-depth interviews were used because they provide rich descriptive data and insight and enabled the authors to explore deeply the experiences and perspectives of women who underwent CS following timely or late family consent [24].

*Study participants*: Women who experienced maternal near miss and underwent CS at the national referral hospital between the 15^th^ of April 2019 and 30^th^ of March 2020 during the data collection for the quantitative part of the study were eligible to be included.

*Sampling*: Purposive sampling was used to select women who had experienced maternal near miss to give deeper understanding information about the phenomenon under investigation [25,26]. This method enabled us to obtain a study sample that provided rich data related to experiences, perspectives barriers, and facilitators of family consent for CS to be performed. A local midwife trained in data collection with the fourth author recruited mothers at the obstetrics department. The fourth author and the local midwife asked the women meeting the criteria of maternal near miss if they wanted to participate in the study. Women who accepted were followed up for in-depth interviews at the hospital. Data saturation was reached after 16 interviews, meaning no new themes or insights emerged with additional interviews. None of the women approached declined to participate. Overall, 16 women who underwent CS participated in the study. The sample was drawn from the quantitative study participants encompassing CS deliveries (n = 16).

*Data collection*: In-depth interviews were conducted in Somali by trained data collectors at the national referral hospital in Somaliland. A semi-structured interview guide was used to collect data. The interview guide contained questions regarding circumstances related to near-miss events, such as, “Please explain your situation and how you experienced this difficulty”; “How was the decision about CS made?”; “Were there delays in the family decision for CS?”; “Who provided the signature for CS?”; and “How long did it take?”. Probing questions were included on barriers and facilitators to family decision making for CS consent. The interviews took approximately 40 minutes and were recorded, transcribed, and translated into English.

*Data analysis*: The analysis of the data in this study followed a deductive thematic approach [26,27]. The preliminary analysis was discussed among the first, second, third and the last author. In the first phase, the first author read the transcripts several times to be familiarized with the data set. The second phase involved generation of codes using the deductive approach of coding the data text in relation to the barriers to and facilitators of family consent for CS to be performed. Examples are shown in Table 1. In the third phase, the codes were examined and re-examined to find repeated patterns, differences, and similarities; these were then organized into themes according to two thematic areas of barriers to and facilitators of family consent for CS to be performed. In the fourth phase, a further check was carried out by mapping the themes back onto the transcripts to ensure that they accurately reflected the original meaning of the text. The fifth and final phase of the process involved further defining and refining every theme and interpreting the data set beyond its original description. Throughout the phases of data analysis other researchers in this study were involved and discussed the analysis back and forth to reach consensus.

**Table 1:** Example of the coding matrix used to identify subthemes according to themes on barriers and facilitators of timely family decision-making for CS to be performed.

Interview transcript	Codes	Sub-themes	Themes
My family and my husband argued for many hours about the decision of my care, but when they accepted the care, my husband and my father together provided signatures. (Interview 5, woman with ruptured uterus)	Family disagreement	Differing understanding between family members on indication for CS	Barriers to timely family consent for CS to be performed
Although my father first disagreed about my operation, after more consultation, he agreed. (Interview 7, woman with eclampsia)	Father disagreed with treatment	Differing understanding between family members on indication for CS	Barriers to timely family consent for CS to be performed
There was a lot of delay because first she refused the caesarean section, then her husband was not available. (Interview 8, woman with eclampsia)	Husband not available in the hospital to give consent	Absence of the person providing formal consent	Barriers to timely family consent for CS to be performed
My husband signed because my family was living in Ethiopia and no one else could stay. (Interview 2, woman with severe APH)	Husband’s independence to give consent	Husband’s autonomous decision-making about CS	Facilitators of timely family consent for CS to be performed
I came from Ethiopia as a migrant, and my husband signed immediately, and there was no delay. (Interview 7, woman with eclampsia)	Husband’s independence to give consent	Husband’s autonomous decision-making about CS	Facilitators of timely family consent for CS to be performed

*Ethical considerations*: Permission to conduct the study was obtained from the Somaliland Ministry of Health Development. Ethical clearance was provided by the research ethics committee of the University of Hargeisa (Dr: CS/41/05/18). The researchers applied the ethical principles and guidelines for research of human subjects. All participants received oral information about the study and provided verbal consent to participate. The consent process was documented in each participant’s study record. This approach was approved by the University of Hargeisa research ethics commitee as part of the ethical review for the study, and all procedures followed were in line with the ethical guidelines and regulations established by the research ethics commitee. They were informed that they had the right to withdraw from the study at any time and that their participation in the study was voluntary. They were also informed that the data would be kept confidential.

## Results

### Quantitative sample characteristics

Of the 516 women recruited for this study, most were aged between 20 and 34 years (64.9%). More than half of these women had no formal education (59.3%). The majority resided in an urban setting (88.6%) (Table 2).

**Table 2:** Sociodemographic Characteristics of Study Participants who underwent CS in the Quantitative Part (n = 516).

Variable	n (%)
**Age**	
< 20 years	87 (16.9%)
20–34 years	335 (64.9%)
> 34 years	94 (18.2%)
**Education**	
No formal education	306 (59.3%)
Formal education	210 (40.7%)
**Residence**	
Rural	59 (11.4%)
Urban	457 (88.6%)

### Qualitative sample characteristics

In total, 16 women who had undergone CS were interviewed. The women’s ages ranged from 22 to 40 years. Table 3 shows characteristics of the women interviewed including their diagnosis and treatment given.

**Table 3:** Characteristics of Study Participants who underwent CS in the Qualitative Study (n = 16).

Interview	Age Group	Level of Education	Diagnosis	Treatment	Family consent timing
Interview 1	20-30	No formal education	Severe antepartum haemorrhage	Blood transfusion of more than 4 units of blood	Late family consent for CS
Interview 2	20-30	No formal education	Severe antepartum haemorrhage	Blood transfusion of more than 4 units of blood	Timely family consent for CS
Interview 3	30-40	No formal education	Severe antepartum haemorrhage and systemic infection	Blood transfusion of more than 2 units of blood and intravenous antibiotics	Late family consent for CS
Interview 4	30-40	No formal education	Eclampsia	Intravenous infusion with magnesium sulphate	Late family consent for CS
Interview 5	20-30	No formal education	Ruptured uterus	Blood transfusion of 5 units of blood and surgical uterine repair.	Late family consent for CS
Interview 6	20-30	No formal education	Eclampsia	Intravenous infusion with magnesium sulphate	Timely consent for CS
Interview 7	30-40	Low level education	Eclampsia	Intravenous infusion with magnesium sulphate	Late family consent for CS
Interview 8	20-30	Low level education	Eclampsia	Intravenous infusion with magnesium sulphate	Late family consent for CS
Interview 9	20-30	Low level education	Severe antepartum haemorrhage, postpartum sepsis	Intravenous infusion with magnesium sulphate and intravenous antibiotics	Late family consent for CS
Interview 10	40-50	No formal education	Eclampsia	Intravenous infusion with magnesium sulphate and blood transfusion of more than 2 units	Late family consent for CS
Interview 11	30-40	No formal education	Severe antepartum haemorrhage	Blood transfusion of more than 2 units of blood	Timely consent for CS
Interview 12	30-40	No formal education	Ruptured uterus	Blood transfusion of more than 2 units of blood	Late family consent for CS
Interview 13	30-40	No formal education	Severe antepartum haemorrhage	Blood transfusion of more than 4 units of blood	Timely family consent for CS
Interview 14	30-40	No formal education	Severe postpartum haemorrhage	Blood transfusion of more than 2 units of blood	Late family consent for CS
Interview 15	20-30	Primary education	Ruptured uterus	Blood transfusion of more than 2 units of blood and surgical uterine repair.	Late family consent for CS
Interview 16	30-40	No formal education	Ruptured uterus	Blood transfusion of more than 3 units of blood and surgical uterine repair.	Timely consent for CS

### Integrating qualitative findings with quantitative results

In this section, the quantitative findings are integrated with the qualitative findings according to the barriers to and facilitators of timely family consent for CS to be performed. The integrated findings, quantitative and qualitative findings are shown in Tables 4–6 respectively.

**Table 4:** Subthemes identified in thematic analysis according to themes on barriers to and facilitators of timely family consent for CS.

Themes	Subthemes
Barriers to timely family consent for CS to be performed	Poor communication and understanding delays informed choice for CS
	Differing understanding between family members on need for CS
	Absence of the person giving consent
Facilitators of timely family consent for CS to be performed	Husband’s autonomous decision-making about CS
	Adequate disclosure of all relevant information about CS

**Table 5:** Integration of quantitative and qualitative findings.

Variable/issue studied	Quantitative finding	Qualitative finding	Converges, diverges, adds, explains, unique, etc.
Barriers to timely family consent for CS to be performed	No formal education (cOR 1.67; 95% CI 1.17–2.39) (aOR 1.15; 95% CI 0.76–1.73)	Poor communication and understanding delays informed choice for CS	Explains
	Hypertensive disorders (cOR 7.82; 95%CI 2.34–26.13) (aOR 8.49; 95% CI 1.08–66.99)	Differing understanding between family members on indication for CS	Explains
	Obstetric haemorrhage (cOR 5.04; 95% CI 1.92–13.22) (aOR 3.21; 95% CI 1.16–8.89)		
		Absence of the person providing formal consent	Adds
Facilitators of timely family consent for CS to be performed	Previous CS (cOR 0.59; 95% CI 0.38–0.87) (aOR 0.73; 95%CI 0.47–1.12)		Adds
	Rural residence (cOR 0.47; 95% CI 0.26–0.85) (aOR 0.54; 95% CI 0.27–1.06)	Husband’s autonomous decision-making about CS	Explains
		Adequate disclosure of all relevant information about CS	Adds

**Table 6:** Background, obstetric, underlying and contributory factors associated with timely family consent about CS.

Variables	Timely family consent for CS ≤ 3 hrs, n = 229 (44%)	Late family consent for CS > 3 hrs, n = 287(56%)	cOR (95% CI)	aOR (95% CI)^a^
**Sociodemographic characteristics**				
***Maternal age***				
< 20 yrs	40 (17.5%)	47 (16.4%)	0.70 (0.39-1.26)	–
20–34 yrs	154 (67.2%)	181 (63.1%)	0.70 (0.44-1.12)	–
≥ 35 yrs	35 (15.3%)	59 (20.6%)	1	
***Parity***				
Nulliparous	41 (17.9%)	55 (19.2%)	0.92 (0.59-1.44)	–
Multiparous	188 (82.1%)	232 (80.8%)	1	–
***Level of education***				
No formal education^b^	120 (52.4%)	186 (64.8%)	1.67 (1.17-2.39)	1.15 (0.76-1.73)
Formal education	109 (47.6%)	101 (35.2%)	1	1
***Place of residence***				
Rural	17 (7.4%)	42 (14.6%)	0.47 (0.26-0.85)	0.54 (0.27-1.06)
Urban	212 (92.6%)	245 (85.4%)	1	1
**Obstetric/underlying and contributory causes**				
***Previous CS (among multiparous)***				
Yes	135 (71.8%)	138 (59.5%)	0.59 (0.38-0.87)	0.73 (0.47-1.13)
No	53 (29.2%)	94 (40.5%)	1	
***Severe Anaemia***				
Yes	20 (8.7%)	13 (4.5%)	2.02 (0.98-4.15)	1.82 (0.82-4.04)
No	209 (91.3%)	274 (95.5%)	1	1
***Obstetric haemorrhage***				
Yes	5 (2.2%)	29 (10.1%)	5.04 (1.92- 13.22)	3.209 (1.16-8.89)
No	224 (97.8%)	258 (89.9%)	1	
***Hypertensive disorders***				
Yes	3 (1.3%)	27 (9.4%)	7.82 (2.34-26.13)	8.49 (1.08-66.99)
No	226 (98.7%)	260 (90.6%)	1	
***Obstructed/prolonged labour***				
Yes	149 (65.1%)	184 (64.1%)	0.96 (0.67-1.38)	–
No	80 (34.9%)	103 (35.9%)	1	

^a^Variables with a p value < 0.2 in the bivariate analysis were included in the multivariable analysis.

^b^No formal education comprises women who have never gone to school and those who have attended Koran School, whereas formal education includes those who have completed primary-, secondary-, or tertiary-level education.

### Barriers to timely family consent making for CS to be performed

#### Poor communication and understanding delays informed choice for CS.

In most of the interviews, women brought to the health facility for obstetric care and their family members initially refused CS. When healthcare providers informed them of the need for CS, it was revealed that they had misconceptions about and low understanding of CS. This was because they preferred a vaginal delivery. They pointed out that lack of adequate education about the indication for CS deterred them from accepting the intervention. The women interviewed expressed the need for effective communication between healthcare providers, family members, and the woman in need of a CS. In this regard, the participants’ accounts of delayed family consent related to inadequate information from healthcare providers and, consequently, a lack of understanding of the need for CS.

*I believed if I underwent CS, my baby would die, I would have constant pain at the surgical site, back pain, and I might easily get an infection; then pain would follow me for the rest of my life, I would not be able to take care of my baby, my milk supply will be affected, and CS would limit my future pregnancies...at that moment, if I got before someone who educated me about CS, I could have accepted early because I realized that I harmed my life due to lack of information. (Interview 15, woman with eclampsia)*

When asked about how consent was sought, many of the women expressed their frustration with not being adequately informed about how healthcare providers arrived at the decision about CS. They mentioned that doctors performed procedures such as obstetric ultrasound and made the decision about CS without informing the woman and her family members on the severe condition of the woman or the foetus. One woman said the following:

*The doctors did not talk to me in a more respectful way, and when I repeated questions or when the family members wanted to make sure the intervention was necessary, the doctors did not answer the questions or re-explain these difficult things they were talking about… my family refused CS, so it took more than three hours, and lastly, they accepted when they realized that I had a very serious situation. (Interview 12, woman with ruptured uterus).* Another woman said,

*The doctor decided to perform CS, and actually I refused. I was angry about the ultrasound that found there was something wrong with my baby, but no one really explained to me what this meant, and I was worried the baby was already dead, or would it die during delivery? I had many unanswered questions, so that is why I refused the care. (Interview 8, woman with eclampsia)*

In the quantitative results, the unadjusted odds ratio showed that women who had no formal education had 1.67 times higher odds of late family consent (crude odds ratio 1.67; 95% CI 1.17–2.39) compared to women with formal education. The adjusted odds ratio showed no statistical significance, indicating that no formal education was not an independent predictor of late family consent. However, the qualitative finding, suggests that a lack of education among women contributes to delayed family consent due to poor understanding and communication about the necessity of CS. The qualitative findings gave a deeper understanding of the complex situation of family consent and that other factors may cause the delay because provision of consent is in the hands of the family.

#### Differing understanding between family members on need for CS.

The quantitative results showed that women with obstetric haemorrhage had higher odds of a late family consent (aOR 3.21; 95% CI 1.16–8.89) compared to women without obstetric haemorrhage. Moreover, women with hypertensive disorders had higher odds of late family consent (aOR 8.49; 95% CI 1.08–66.99) compared to women without hypertensive disorders. Consent for CS was given late despite the maternal complications. This quantitative finding was consistent with qualitative results.

The participants who were interviewed pointed out that it was time consuming for families to consult on the care indicated by healthcare providers. Moreover, families argued with each other before agreeing for CS to be performed due to a lack of trust in the healthcare and the doctors’ judgement. Family disagreements were said to result due to differences in opinions between family members on what constituted the best care after doctors proposed that CS should be performed. Some of the family members preferred other modes of delivery rather than CS, even though their significant other had a severe maternal complication. One of the women said, “My father believed it was very important to give birth at home with a normal delivery, and hence, he didn’t want any more hospital involvement and treatment” (Interview 7, eclampsia). Some of the women interviewed mentioned that some of their family members held the view that CS was unnecessary and that the woman would be able to give birth normally. Other family members had accepted CS as a mode of birth that could resolve the woman’s complication. It took time for them to have discussions with the family members to agree on giving consent. One woman said,

*Both my sister and my husband refused to give consent for Caesarean section since I had prolonged labour within two days. They believed that I could give birth naturally, so that he went to town and switched off his phone, and my mother called him many times, but he could not respond. Although he accepted finally, but it took to provide consent after 2 days to beg him, and later he consented, and the doctor performed CS. (Interview 14, woman with severe postpartum haemorrhage)*

Nonetheless, some of the women stated that their family members understood the severity of their condition and agreed to provide consent. However, other family members refused to provide consent. They were not convinced that CS was the appropriate care. In this regard, differences in understanding between what families found to be the appropriate care resulted in delayed family decision-making.

*My husband was ready to provide the signature, although we were shocked about this. However, my family refused to let my husband to sign because my family was not confident about the Caesarean section, and it took me to wait within two additional hours; after more consultation, my family allowed, and lastly, my husband signed. (Interview 9, woman with severe antepartum haemorrhage and postpartum sepsis)*

One woman who experienced obstetric haemorrhage due to ruptured uterus said, “Midday of the 11^th^ day, the doctor decided on caesarean section, and around 6-7 hours, my family and relatives argued about the decision of my care, whether to agree to caesarean section or not, but lastly, they accepted when they realized my severity” (Interview 5, woman with ruptured uterus).

Another woman said,

*Although my husband was ready to sign, my mother was very upset and refused him to sign for my CS; after two hours, my father convinced her that she should get out of this and accept his decision to sign as maybe otherwise I would die too like the baby. Then she accepted.* (Interview 3, woman with severe antepartum haemorrhage and systemic infection)

The qualitative findings help explain the quantitative results, particularly the unexpected association between hypertensive disorders and late family consent. Although women with hypertensive complications require urgent care, the interviews revealed that family members often misunderstood the severity of such conditions and spent considerable time debating whether CS was necessary. These differing perceptions, coupled with mistrust in healthcare providers, contributed to delayed consent even in clinically urgent situations.

#### Absence of the person providing formal consent.

The qualitative finding on absence of the person giving consent was an additional finding to the quantitative results. This finding gave a deeper understanding of the barriers to timely family consent. The participants who were interviewed mentioned that when healthcare providers informed them that CS should be performed, the person with the authority to give consent was not available at the hospital to provide consent. For this reason, consent could not be given on time because it was time consuming for the person to come to the hospital to give consent.

The women interviewed stated that when the person who was supposed to provide consent was not available, it further delayed performance of CS because delays had already been experienced due to the initial refusal by the woman indicated for CS. In addition, when another family member was called in to provide consent because the husband was not available, the family member declined to provide consent. One woman said the following:

*Two days later, I agreed, but my husband was still not found, and my brother-in-law was brought to the hospital in hopes that he could take responsibility and sign on his brother’s behalf, and also, he refused to sign the consent as he was worried his brother would get angry with him. My husband came and consented on the 5*^*th*^ *day after admission, and the Caesarean section was performed.* (Interview 10, woman with eclampsia)

*Really there was lot of delay because first i refused the c/section and other care, and it took to accept around ten hours; then, when i accepted, my husband was not available, so i called her father to sign, and when, he arrived he signed for my care.* (Interview 8, woman with eclampsia)

Some of the other women interviewed explained that they had to contact their fathers, to provide consent for CS to be performed. However, when the family members were contacted, they initially refused to sign the consent for the operation to be performed. This contributed to further delays in timely provision of consent for CS. One of the women said, “My father gave signature to my operation because my husband was not available, although my father refused first, but later he obeyed...when the condition became serious and my baby died” (Interview 12, woman with ruptured uterus).

### Facilitators of timely family consent for CS to be performed

#### Husband’s autonomous consent about CS.

In the qualitative interviews, participants explained that in cases whereby the husband was alone when healthcare providers requested consent, he provided consent without delays. In these instances, the in-laws were not present at the healthcare facility. This enabled the husband to have full independence in making a timely decision for CS to be performed. This finding meant that when there was limited involvement of other family members in the decision-making process when CS was indicated, the husband provided consent early, without delays, and had full authority.

One woman said, “My husband signed because my family lived in Ethiopia and no one of my family could stay, so he had full authority to give the signature for my operation” (Interview 2, woman with severe antepartum haemorrhage).

In addition, some of the women stated that their husbands proceeded with giving consent immediately after informing the family that their wives were undergoing CS. In these cases, the process of seeking consent was not time consuming as the husband did not have extensive discussions with family members prior to giving consent. Husbands, in this regard, had more control over the decision-making about CS.

One woman said, “When it comes to the signature, my husband was with me and accounted for my care, and he suddenly told my family and continued to sign rapidly because I was severely sick and I had massive bleeding” (Interview 2, woman with severe antepartum haemorrhage).

The quantitative results also sought to investigate the role of place of residence in timely family consent about CS. The unadjusted odds ratio showed that women from rural areas had 0.47 lower odds of late family consent for CS (cOR 0.47; 95% CI 0.26–0.85) compared to women from urban areas. In the qualitative findings, some of the women who were brought to the health facilities from the rural areas came with their husbands. The presence of the husband only or fewer family members at the healthcare facility when consent was requested by healthcare providers contributed to timely family consent. One woman said, “I lived in Daami Village...but it took me to travel to the hospital around one hour. My husband and my friends stayed with me so that as soon as the doctor decided on an operation, he signed, and there was no delay” (Interview 6, eclampsia). Another woman said,

*It was very long journey from Moolli village in Gashaamo near the border of Ethiopia, because I left home at 3am and came to Hargeisa at 4 pm since the car went straight because I was severely sick, and my husband encouraged the driver to speed up...my family and my husband provided the consent since my baby was dying; my family agreed, and consent was provided. (Interview 13, woman with severe antepartum haemorrhage)*

However, the adjusted odds ratio showed no statistical significance between place of residence and late family consent for CS. The qualitative finding on fewer family members from rural areas during decision-making about CS gave a deeper understanding of the quantitative results on rural residence as a facilitator of timely family consent.

#### Adequate disclosure of all relevant information about CS.

When the family received more information regarding the possible risks and bad outcomes of not giving consent early, consent was given on time without delays. Participants also pointed out that when the responsible doctor explained and engaged in a discussion related to the care provided, there was timely family consent. One woman said, “Although my father first disagreed [about] my operation, after more consultation, he agreed” (Interview 7, woman with eclampsia).

*The health care providers told him to sign consent for my care in order to save my life because my situation was very serious, and he did not have time to take other options since I had more bleeding, and they told him the only way to save my life was caesarean section. My baby died early as vaginal bleeding started, and also my situation was severe, and it was mandatory for my husband to consent to my operation. (Interview 2, woman with severe antepartum haemorrhage)*

Some of the women who survived their complications and gave birth to a healthy baby following CS emphasized the importance of explaining adequately to family members about the complications that the women were having. One woman said, “Although I and my baby survived the problem, when women experience any difficulties, they might suffer many times because the relative did not know what happened or any complication that arose, so that it is better to tell the families what is the cause of complication” (Interview 14, woman with severe postpartum haemorrhage).

The qualitative finding on adequate disclosure of all relevant information about CS was not in the quantitative results. However, this finding provided a deeper understanding about what facilitates a timely family consent.

#### Previous CS.

The quantitative results showed that women who had had a previous CS had higher odds of a timely family consent CS (cOR 0.59; 95% CI 0.38–0.87) compared to women who had not had a previous CS. However, there was no statistical significance in the adjusted odds ratio. There was no qualitative finding that integrated with the finding about previous CS.

## Discussion

To the best of our knowledge, this study is the first to explore the barriers to and facilitators of timely family consent using a mixed-methods approach in Somaliland. Our findings provide vital evidence on the importance of ensuring that families have a deep understanding of the reasons for performing CS in order to provide timely consent for CS. In particular, our main findings showed that poor communication and understanding delayed informed choice for CS, differing understanding between family members on the need for CS and absence of the person giving formal consent contributed to delays in timely family consent. Having had previous CS, the husband’s autonomous consent about CS, and adequate disclosure of all relevant information about CS contributed to timely family consent. Contrary to our previous understanding, residence in rural areas was also associated with timely family consent.

Our study showed that poor communication and understanding delayed informed choice for CS. Although the consent for CS is provided by the family members, it is possible that lack of formal education among most of the women delayed understanding about the need for CS and timely family consent. In line with previous studies, women’s lack of formal education contributed towards delays in obtaining informed consent for CS to be performed [28,29]. However, our findings showed that a lack of formal education was not an independent determinant of timely family decisions in the multivariable analysis. It is possible that other influential factors, such as the family members understanding and trust in health professionals’ judgement, played an important role in decision-making for CS because provision of consent for CS was in the hands of the family.

Moreover, differing understanding between family members on the need for CS led to late family consent to CS. This finding is consistent with previous studies that family conflicts, disagreements, and contradictory advice on the care proposed by doctors can lead to delays in provision of obstetric care [30,31]. In our study, despite women having severe maternal complications, family members took time to give consent for CS due to delays in arriving at a consensus on the recommended mode of birth. Contrary to our expected findings, women with obstetric haemorrhage and hypertensive disorders had higher odds of late family consent compared to those without obstetric haemorrhage or hypertensive disorders. This finding showed that family members possibly preferred other alternative methods of birth rather than CS, even when the woman was in a critical state. Qualitative findings from our study showed that families struggled to grasp the severity of such complications, perceiving them as conditions that could still be managed medically without surgical interventions. In some cases, family members expressed fear of surgical risks or the belief that caesarean section was a last result leading to prolonged discussions and delayed consent. These insights highlight how limited understanding of obstetric complications, combined with sociocultural perceptions about childbirth and medical interventions, may reduce perceived severity and susceptibility, leading to delays in decision-making during emergencies [32].

Late family consent in our study was also caused by absenteeism of the person required to give consent for CS. In the study setting, it is not the woman herself who gives consent for CS [8]. In this regard, although the husband’s autonomous decision making was a facilitator of timely family consent for CS to be performed, their absence jeopardized the health condition of the woman in need of CS. This is because when required to provide consent in an urgent situation, they were not available to do so. The absence of family members, such as the husband, at the hospital during childbirth and when husbands are required to give consent [33], has been previously found to increase delays in consent about CS [34,35]. However, the husband’s autonomous decision-making about CS explained the unexpected finding on higher odds of timely family consent among women residing in rural residences. Rural residence was a significant predictor of timely family consent in the crude logistic regression, but this effect was lost in the multivariable analysis, indicating that family members and healthcare providers influenced timely consent about CS. Our results show the importance of male involvement during counselling and when healthcare providers are requesting CS consent [35].

We found that the initial communication of healthcare providers was inadequate and did not provide sufficient information for family members to provide early consent. Our finding is similar to findings from previous studies that emphasized the importance of effective communication when providing care to women during childbirth [36–38]. One of the studies showed that possible reasons for poor communication were inadequate healthcare providers’ knowledge and skills in communication, forgetfulness, and assumptions about women’s knowledge (that the obstetric procedures were known to them) [36]. Our finding shows the importance of effective communication between healthcare providers and the family of the woman undergoing CS. Effective communication means that all parties involved with the consent process are able to listen to each other and fully understand what is being said [39]. In-depth explanations by healthcare providers about the indications for CS can improve the understanding of family members, as well as women undergoing CS, to obtain consent on time. In contrast, adequate disclosure of all relevant information about CS was a facilitator of timely family consent. Although limited studies have been conducted examining the association between adequacy of informed consent and timely decision-making about CS, this finding is consistent with previous studies that have shown the importance of providing adequate information on the benefits and risks of CS in order to ensure that women and family members provide informed consent [28,29,40,41].

Educational interventions focusing on antenatal care and male involvement within a shared decision-making process [42] can provide an opportunity to increase awareness and prepare husbands to provide early CS consent during childbirth if it is required.

In Somaliland, the process of enacting a policy allowing women to give consent in maternal health emergencies has encountered significant challenges, reflective of broader cultural norms and ethical considerations [5]. While the principle of autonomy underscores the importance of individuals being able to make informed decisions about their own healthcare [43], especially in critical situations like maternal health emergencies, cultural traditions in Somaliland prioritize collective decision-making. This tradition extends to healthcare settings, where even men are not typically allowed to provide consent for surgical procedures without the involvement of male family members. This cultural context creates a complex ethical dilemma, as it highlights the tension between respecting cultural traditions and upholding individual autonomy and women’s rights [44]. The stalled enactment process not only impacts women’s ability to make timely and informed decisions about their healthcare but also raises broader questions about gender equality and reproductive rights in Somaliland. Further exploration of these ethical dimensions on medical ethics, women’s rights, and cultural anthropology, is essential for understanding the implications of the lack of maternal emergency policy and the challenges it presents in promoting women’s health and rights in the region.

### Strengths and limitations of the study

One of the major strengths of this study is the use of a mixed-methods, convergent design, which enabled findings from quantitative and qualitative data to be compared and contrasted with each other. The method led to a comprehensive understanding of the barriers to and facilitators of timely family decision-making. Another strength of this study is data quality assurance, whereby the first author conducted regular data checks to identify and eliminate any missing quantitative data. The trustworthiness of the qualitative part of the study was increased through inclusion of researchers from different disciplinary backgrounds. This helped prevent the first author from being biased and influencing the findings. In this regard, researchers were involved in regular discussions during data analysis. To enhance confirmability and dependability, an audit trail was kept consisting of audio recordings and notes on coding and data analysis. Transferability was ensured by including a rich description of the study setting, study participants’ backgrounds, and procedures for recruitment.

A limitation of our study is the exclusion of male family members, particularly husbands, whose involvement in decision-making may influence the timing and process of family consent for cesarean section. Future research could consider including the perspectives of male family members by conducting mixed-gender interviews or organizing separate focus groups for men to better understand their roles and views in the decision-making process. This would provide a more holistic view of family consent dynamics.

Another limitation of our study is the use of a > 3-hour cutoff to define late CS, which may not account for clinical context and individual patient circumstances. This cutoff was chosen based on existing literature [12], but it may have excluded cases where delays were clinically justified. Alternative cutoffs, such as considering a delay of more than 30 minutes after the doctor’s decision, were not considered, as we lacked data on the clinical indication for CS, which could have informed the acceptable delay. The lack of this data may limit the generalizability of our findings, as we cannot ascertain whether the delays were clinically appropriate. Future studies should include information on CS indications to refine the definition of “late CS” and explore how these delays affect maternal outcomes.

The absence of data on the indications for CS is another significant limitation, as it is crucial to understanding the urgency and appropriateness of delays in surgery. Without this information, we may risk overgeneralizing the findings or missing important nuances in the decision-making process. In future research, it would be important to collect data on the clinical reasons for CS to provide a fuller context for assessing delays in surgery and their potential impact on maternal and neonatal outcomes. Although conducting interviews in Somali, a language well understood by the participants, was a strength that enhanced the depth and authenticity of the data, the translation from Somali to English might have led to some loss of meaning [45]. To mitigate this, transcripts were rechecked by the fourth author and bilingual validation was conducted to ensure the preservation of the original meaning. This additional step further validated the reliability of the qualitative data.

## Conclusion

This study identifies poor communication between healthcare providers, women in need of CS, and their families as a key factor contributing to delays in obtaining family consent for CS. A lack of understanding among family members about the necessity of CS led to delayed decisions. Effective disclosure, particularly regarding the husband’s role in decision-making and the impact of previous CS, plays a crucial role in timely family decision-making.

We recommend that healthcare providers receive specialized training to enhance communication during consent processes, particularly around informed consent for CS. A standardized counseling package should be developed for healthcare providers, including education for husbands and male family members. This could be implemented starting during antenatal visits and continuing through childbirth to ensure timely, informed decision-making.

Additionally, we propose empowering women with greater autonomy in making health decisions, including giving consent for emergency procedures. This can be achieved through a participatory, collaborative policy-making process involving women, men, religious leaders, healthcare providers, and policymakers. Such policies would support women’s rights to make independent decisions during maternal health emergencies.

### Implications for future practice

This study provides actionable insights that bridge research and practice, guiding healthcare providers, policymakers, and institutions in improving clinical decision-making. Strategies to address delays in family consent such as involving male family members and enhancing communication during emergencies can directly improve patient care and outcomes. The findings also have implications for policy development, including streamlining consent procedures, developing culturally sensitive communication strategies, and ensuring clear protocols for timely CS.

Additionally, the study highlights the need for training healthcare professionals to navigate cultural and familial dynamics, improving capacity in both clinical and community settings. Addressing identified gaps, such as the exclusion of male perspectives and CS indication data, will help create more inclusive practices. Understanding barriers to family consent can also improve community health education programs, fostering trust and collaboration between families and healthcare providers.

Finally, this study aligns with Sustainable Development Goal (SDG) 3, which focuses on reducing maternal and newborn mortality. By emphasizing the provision of timely cesarean sections, the study contributes to efforts to improve maternal health outcomes and highlights the importance of informed, timely decision-making in reducing preventable deaths. This alignment underscores the study’s broader impact, offering a foundation for future research, policy initiatives, and community engagement to enhance maternal health.

## Abbreviations

CS: Caesarean section.
